# Semantic centrality and emotional valence contribute to word memorability in an associative memory task for Chinese words

**DOI:** 10.1038/s41598-026-37531-w

**Published:** 2026-02-25

**Authors:** Zhang Allen Haoyu, Wilma A. Bainbridge, Pei Sun, Andy C. H. Lee

**Affiliations:** 1 Department of Psychology (Scarborough), University of Toronto, Toronto, Canada; 2 Department of Psychological and Cognitive Sciences, Tsinghua University, Beijing, China; 3 Department of Psychology, University of Chicago, Chicago, USA; 4 Neuroscience Institute, University of Chicago, Chicago, USA; 5 Faculty of Health and Wellness, City University of Macau, Macau, SAR China; 6 Rotman Research Institute, Baycrest Academy for Research and Education, Toronto, Canada

**Keywords:** Word memorability, Semantic knowledge, Emotion, Associative memory, Semantic centrality, Neuroscience, Psychology, Psychology

## Abstract

Memorability, an intrinsic property of stimuli, reflects the consistent likelihood of a stimulus being remembered across individuals. Recent research has examined English word memorability in an associative memory task and found that the overall semantic connection of a word to other words (termed *semantic centrality*) predicts its memorability, with stronger semantic centrality associated with a greater likelihood of successful recall. However, it is unclear whether these findings apply to other languages. Moreover, whether and how emotional content contributes to word memorability has not been fully explored. Of particular interest is emotional consistency: whether emotional words that are of the same or different valence (e.g., positive with positive vs. positive with negative) exhibit a mnemonic advantage over neutral words in associative memory. To address these issues, we examined semantic centrality and emotional consistency as predictors of memory performance in three visually-presented cued-recall experiments using Chinese words. Participants encoded pairs of nouns and were then required to recall target words in response to cue words. We found that both semantic centrality and emotional consistency contributed significantly to word memory: surprisingly, and in contrast to previous work in the English language, words with lower semantic centrality were associated with higher memorability; broad emotional consistency (non-neutral cue-target pairs) and positive emotional consistency (positive-positive pairs) both exhibited memory advantages. Notably, our two factors of interest did not interact with each other and demonstrated additive effects when explaining memory performance. Our results provide insights into word memorability, specifically as it relates to the Chinese language, and potential contributions of semantics and emotion.

**Supplementary Information:**

The online version contains supplementary material available at 10.1038/s41598-026-37531-w.

## Introduction

Memorability refers to an intrinsic stimulus property that captures how likely a stimulus will be remembered after it has been encountered^1^. Empirical research has demonstrated and validated stimulus memorability across multiple domains of stimuli, including images^2,3^, faces^4–6^, words^7–9^, artwork^10^, and voices^11^. In other words, memorability can explain substantial variance in what we remember and forget.

One line of ongoing research is whether and how memorability is connected with or explained by other stimulus attributes^12^. In particular, previous studies have highlighted the contributions of semantic properties to word memorability, including animacy^13,14^, ambiguity^15,16^ and the degree to which a word is semantically related to others^9^. Concerning this last property, Xie et al.^9^ built a computational model of a semantic network that comprises words as the elements and the degree of semantic relatedness as the connections between the elements. According to this model, words that are more strongly connected (i.e., are more semantically related) are more likely to be recalled during cued recall (i.e., possess higher memorability). This modelled semantic attribute, originally termed semantic memorability (but referred to as semantic centrality from here on) , aligns with theories of probabilistic search of associative memory, which suggest that retrieval of a target word relies on its connection with contextual cues^17^. It is supported by both behavioural and neurological data^9^ and is consistent with the idea that the human brain represents the relatedness among semantic concepts^18–21^. Specifically, Xie et al.^9^ found that participants had higher cued-recall probability for words with higher semantic centrality, and that when recalled, words with high semantic centrality were reinstated faster in the anterior temporal lobe than words with low semantic centrality, as shown by intracranial recordings.

The aforementioned work raises two intriguing questions. First, it is unknown to what extent existing findings pertaining to semantic centrality and word memorability generalize to cultures and languages beyond Western culture and English, such as East Asian culture and Chinese. Memorability is implicitly assumed to be cross-culturally consistent, supported by empirical evidence of face memorability consistency between US and South Korean samples^22^. However, since words are embedded within language systems that differ across cultures, the semantic mechanisms underlying word memorability may vary as well. Chinese differs from English in its lexical structure, particularly in its heavy reliance on compounding morphology where words are formed by combining meaningful components (i.e., morphemes). This strong semantic compositionality may facilitate associative encoding through novel combinations^23^, even for words with low semantic centrality, thereby moderating the influence of semantic centrality on their memorability. In addition, cross-cultural differences in cognitive processing may influence memory encoding: East Asian samples tend to engage in holistic, relation-based encoding, whereas Western samples prefer analytic item-focused processing^24^. Notably, prior work in other domains has shown that distinctiveness can enhance memorability^4,25^, suggesting that semantic influences on memory are not necessarily uniform. Based on these considerations, two alternative predictions arise. If word memorability is influenced by a universal organization of semantics, the effect of semantic centrality observed in English should also emerge in Chinese. Conversely, if memorability is modulated by language-specific semantic structure or culture-related cognitive biases, the influence of semantic centrality could differ in magnitude or even in direction across languages. Taken together, the inclusion of Chinese enables a boundary test of the role of semantic centrality in associative memory.

Second, the potential impact of emotion on word memory is not clear, an important issue given that emotional content has been previously shown to influence performance on word memory tasks^26–32^. For instance, compared to neutral words, negative words were found to elicit better item recall^29^, while positive words were better recalled in an associative memory task^30^. One underexplored issue is whether the emotional consistency of word memoranda enhances memory recall, especially cued recall. Word emotional consistency captures whether two (or more) words belong to the same valence category (i.e. positive, negative, and neutral) and it can be independent of semantic centrality. For instance, two positive words that are emotionally consistent can be either semantically similar (e.g., ‘champagne’ and ‘wedding’) or distant (e.g., ‘champagne’ and ‘serenity’). Two studies that have investigated this topic and focused on positive-positive pairs^30,33^ reported that positive word targets paired with positive cues are better recalled compared to neutral-neutral target-cue pairs, as well as positive-neutral and negative-negative target-cue pairs. A natural follow-up question is whether a broader form of emotional consistency exists, in which emotional information is represented at a binary level (emotional vs. neutral) independent of valence polarity. Under this account, a word pair would be considered emotionally consistent whenever both items carry emotional significance, even if they differ in valence (e.g., positive-negative). This possibility is theoretically meaningful because it would imply that emotional coherence in associative memory may arise from shared emotional salience rather than shared valence direction. Surprisingly, no prior study has directly tested this question, despite its theoretical importance for associative binding models^31^. Further, if emotion does indeed have an impact on word associative memory, two interesting questions are whether its effect remains significant when semantic centrality is controlled and whether the emotional consistency effect complements or interacts with that of semantic centrality.

Here, we implemented three cued-recall experiments to study the contributions of semantic centrality and emotional consistency to Chinese word memory. In the cued-recall paradigm, a cue word is presented during retrieval to prompt recall of its paired target^9^. Unlike free recall, where words must be retrieved without any lexical cue, cued recall explicitly encourages associative encoding and retrieval, thus constituting a fundamentally different memory process. While our primary focus is on target memorability, this paradigm also permits exploratory consideration of the potential contribution of cue characteristics to memory performance. In Experiment 1, we first used neutral Chinese nouns to examine whether previously reported findings pertaining to semantic centrality in English^9^ generalize to a language other than English. A pool of positive, negative, and neutral Chinese words was then used in Experiments 2 and 3 (the latter a replication of the former) to explore the effect of emotional consistency and its potential interaction with semantic centrality on word memory performance. Consistent with previous findings ^9,30,33^ we hypothesized that semantic centrality and emotional consistency would enhance the memory performance for Chinese words. We predicted that the effect of emotion would persist even when semantic properties are controlled for^26,34,35^ (however, see^36,37)^ but remained open as to whether semantic centrality and emotional consistency would interact in their impact on Chinese word memory. To the best of our knowledge, our work provides novel insight into the impact of semantic centrality, emotional consistency, and their interaction on word memorability.

## Experiment 1

### Methods

#### Participants

Fifty-nine participants (thirty-one females, mean age = 21.14, SD = 2.49 years) were recruited from the student population at Tsinghua University and received monetary compensation for participation. The sample size was determined a priori to be consistent with previous research on word memorability using cued-recall tasks (sixty in Madan et al.^30^; thirty with two sessions per participant in Xie et al.^9)^. To further evaluate statistical sensitivity, we conducted a simulation-based post-hoc power analysis using the *mixedpower* package in R^38^, and the results are reported in Table S1. All participants had Mandarin Chinese as their singular first language and self-reported to have no historical or current mental and/or neurological disorders. Prior to the experiment, participants signed an informed consent form. The study was approved by the local Ethics Committee at Tsinghua University. All procedures were performed in accordance with the relevant guidelines and regulations and with the Declaration of Helsinki. One participant’s data were lost due to a technical fault, and all data from the other fifty-eight participants (thirty females, mean age = 21.09, SD = 2.48 years) were included in the statistical analyses. Data, analysis codes, and stimuli for all experiments are available at osf.io/7bhgk/.

#### Materials

Three hundred words written in Simplified Chinese were selected as stimuli, matching the number of English words used in Xie et al.^9^. Two-character nouns, rather than single characters, were used as stimuli since most single Chinese characters do not have a specific meaning on their own and can have divergent meanings when paired with another character. For instance, the character 礼 forms one-half of the Chinese noun 婚礼 (wedding) as well as the noun 葬礼 (funeral).

Words were selected from the classic MEgastudy of Lexical Decision in Simplified CHinese (MELD-SCH) database^39–43^, which contains 1,985 two-character nouns and provides ratings of valence, arousal, abstractness, and frequency as well as the age of acquisition and the number of strokes of each word. We reversed the abstractness ratings, which are on a 5-point Likert scale, to give us a concreteness measure for our analyses (i.e., concreteness value = 6 – abstractness value). Three hundred neutral words were subsequently selected as follows: first, all words that were outliers on any one of the aforementioned properties (i.e., beyond 3 SD of the mean) were excluded; and second, of the remaining word pool, words that possessed a valence rating higher than 3 and lower than 5 (on a 7-point Likert scale) and were of medium to high frequency (log-transformed mean frequency = 2.84, SD = 0.37, min = 2.05, max = 3.88), and high concreteness (mean = 4.61 out of 5, SD = 0.15, min = 4.35, max = 4.96), were chosen. To ensure comparability with previous memorability research that relied on concrete words in English^9^, Experiment 1 used Chinese words with high concreteness. Moreover, each item was manually checked to ensure a direct and unambiguous English translation.

Since there were 44,850 possible word pairs, no two participants saw the exact same set of word pairs, and the word pairs were randomly determined. Including both practice and main experiment, all 300 words were used exactly once, either as a cue or a target. With a set of fifty-eight participants, each word was a target 25.13 times in the main experiment on average (min = 14, max = 37 times).

#### Procedure

**Fig. 1:**
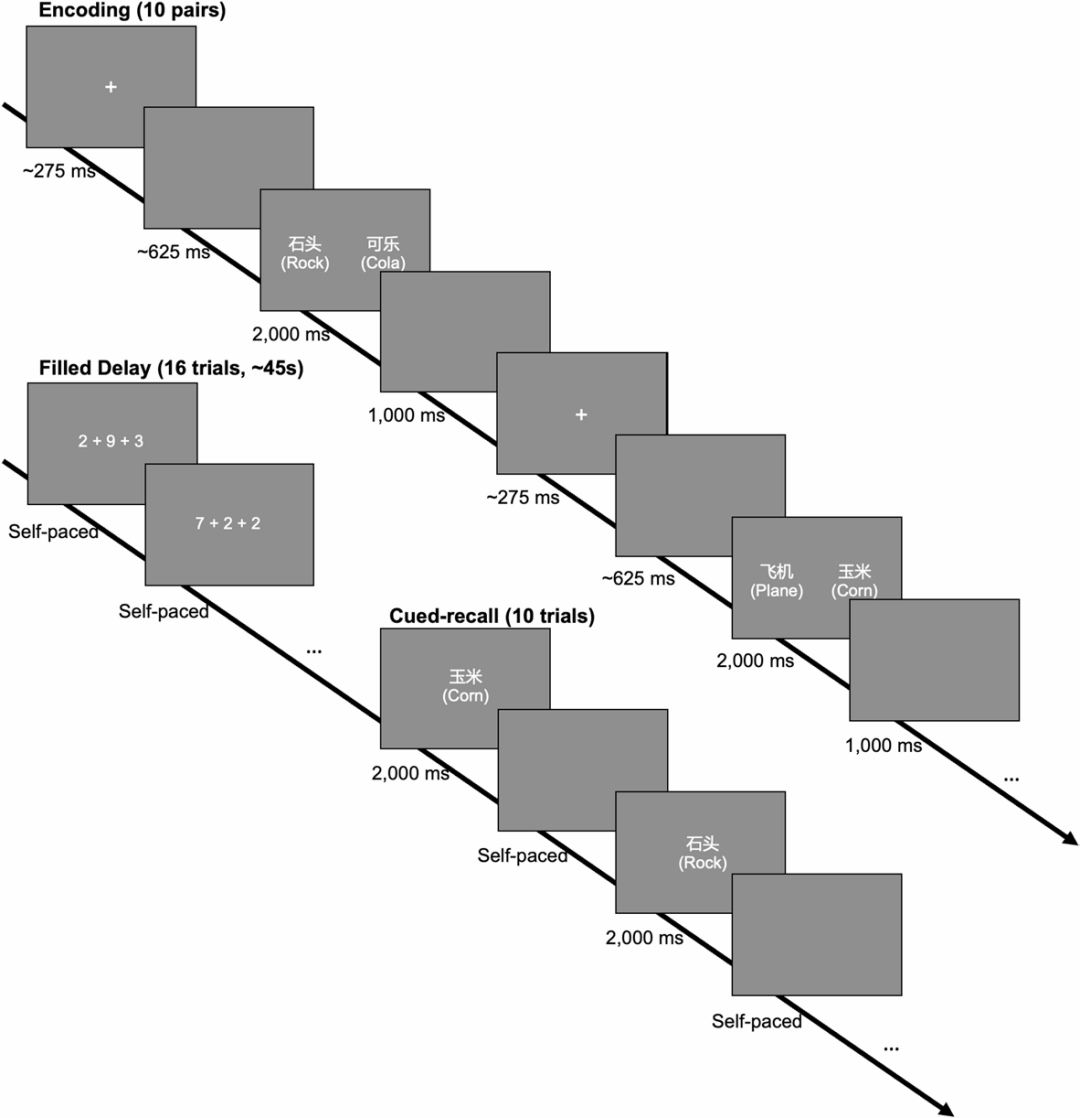
The paradigm in Experiment 1. English words shown in the brackets above are translations of the two-character Chinese noun stimuli used.

Participants were administered thirteen blocks of a paired associate memory task on a desktop computer with a 27-inch display (2560 × 1440 resolution). Each block was composed of an encoding phase, a filled delay, and a cued recall phase (Fig. 1).

In the encoding phase, participants were instructed to remember 10 random pairs of two-character neutral nouns. The word pairs were presented sequentially for 2,000 ms, each in white in the center of a gray background, one word on the left and the other on the right of the central point. Each word pair was preceded by a jittered fixation screen (250-300 ms, mean 275 ms) and a jittered blank screen (500-750 ms, mean 625 ms). After each word pair’s presentation, there was a blank inter-stimulus interval (ISI) of 1,000 ms.

After the encoding phase, an arithmetic task was then administered as a brief filled delay, or in other words, a distraction task. Participants were instructed to type the answer to sixteen questions requiring the addition of three single-digit natural numbers (e.g., “2 + 7+4”). This task was self-paced and took approximately 45 s on average.

In the cued recall phase, participants were administered 10 self-paced trials in which one word from a previously presented word pair was shown on each trial and participants were asked to recall its counterpart by typing it out. Specifically, as is standard procedure when typing Chinese, they typed out the PinYin - the standard romanization phonetic system for Mandarin Chinese - and then selected the appropriate character from among the options provided on screen. None of the words used in the experiment had the same PinYin, although 1.2% of all possible word pairs shared a common character. Each word cue was presented for 2,000 ms and participants could continue to type after the cue disappeared. A blank 1,000 ms ISI started once the participants had made their response and pressed the ENTER key. Word cues were selected randomly from the preceding encoding phase and presented in random order.

Participants received detailed instructions, confirmed their understanding of the task, and were administered two practice blocks before the start of the experiment. The whole experiment took around 45 min in total.

### Data analysis

All analyses were conducted in MATLAB 2020b and R 4.1.3.

#### Consistency of word memorability observed across participants

In the current and all subsequent analyses, we focused on target word memorability as the primary factor, consistent with prior work on word memorability in cued recall tasks^9^. Throughout the manuscript, *memorability* refers specifically to target memorability unless otherwise noted. Cue memorability was additionally examined exploratorily to assess whether retrieval success also depends on the properties of the cue item. First, we analyzed the consistency of word memorability observed across participants, using a split-half consistency check^9,44,45^. Across 5,000 iterations, the participants were randomly split into two halves. The probability of successful cued-recall was calculated for each target word (across many different cues) for each split-half and these values were then used to derive a Spearman’s Rho rank-correlation coefficient between words’ memorability in each pair of split-halves. A 95% confidence interval (CI) was then calculated from the 5,000 coefficients, one for each iteration, with a positive 95% CI reflecting consistent memorability across participants. As a comparison, we repeated this analysis but randomly shuffled the word labels for one split-half group prior to running the Spearman’s correlation for each iteration. We computed the corresponding 95% CI for the shuffled data. We additionally explored whether cues have a similar construct of memorability in associative memory. To this end, the same aforementioned analyses were implemented for the cues, in which the successful recall probability associated with each cue, rather than each target, was calculated.

#### Modeling semantic centrality

**Fig. 2:**
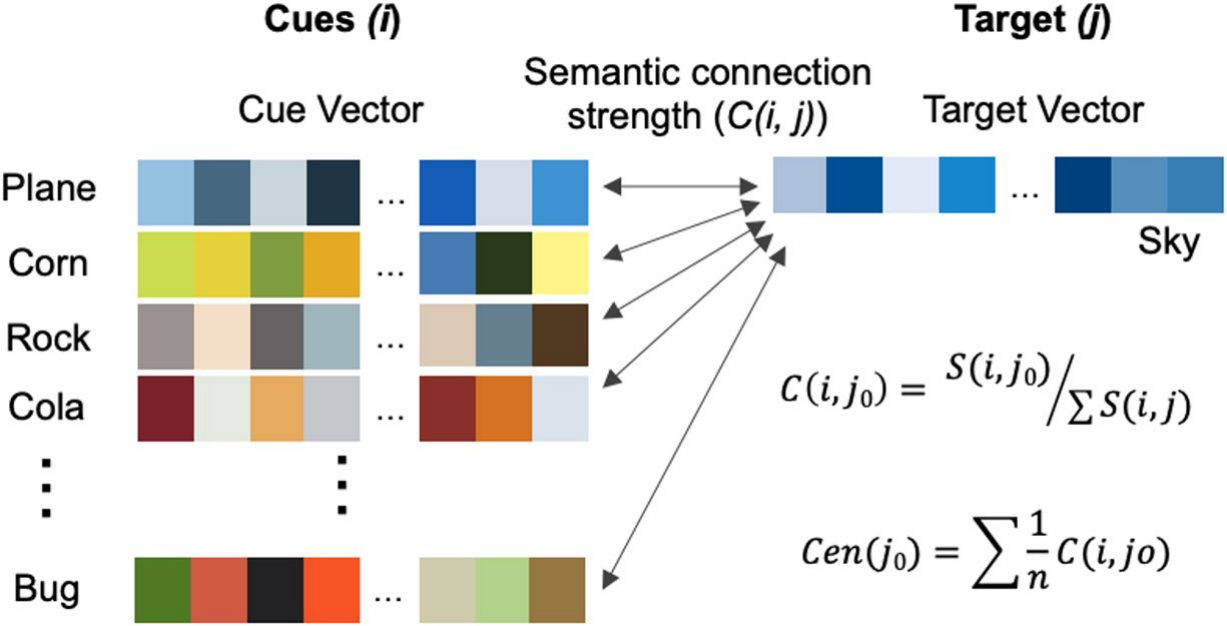
Modelling semantic centrality. *S(i*,* j*_*0*_*)*: raw semantic similarity between a cue (i) and the target (j_0_), derived from the cosine distance metric of the two word embeddings; *C(i*,* j*_*0*_*)*: semantic connection strength between a cue and the target, measured by their semantic similarity relative to the similarity of other words (i.e., potential distractors) to this cue; *Cen(j*_*0*_*)*: the semantic centrality of the target, defined as average semantic connection strength to all possible cues. The colour coding is used here as a heuristic illustration of how similarity between words can be conceptualized, whereas in our analyses the actual similarity values were computed using 300-dimensional numerical vectors.

Following previous work on English word memorability^9^, we modeled a theory-driven construct of semantic centrality. Importantly, semantic centrality was derived independently of the current experimental data, ensuring that it reflects an intrinsic stimulus property rather than task- or sample-specific performance. Consistent with Xie et al.^9^’s theoretical framework and empirical findings, the assumption of this model was that words with stronger connections to other words exhibit higher semantic centrality. We modelled the semantic centrality for each word in three steps (Fig. 2). First, we calculated the semantic similarity (*S(i*,* j)*) between every possible pair of words from our 300-word stimulus set using the word2vec model^46^ for Chinese words^47,48^. This model represented every word as one 300-dimension embedding in the semantic network, with the embeddings trained on the Baidu Encyclopedia corpus, and computed the cosine distance between every two semantic embeddings as a proxy for their semantic dissimilarity. Semantic similarity was then defined as the inverse of this value (1 – dissimilarity). We selected Baidu Encyclopedia because it offers broad, domain-general semantic coverage and serves as the closest Chinese counterpart to Wikipedia, which was used in prior English semantic centrality research^9^. As estimated from a statistical model of language use, words that tend to occur in similar contexts receive higher semantic similarity values. Next, we used these semantic similarity values to determine the likelihood of retrieving the correct word in response to a given cue. This probability was the equivalent of the target’s strength of semantic connection to the cue relative to all potential targets’ connection strength to the cue (*C(i*,* j)*) and was calculated by dividing the cue-target semantic similarity by the sum of the semantic similarity between the cue and all words (other than the cue itself). Finally, we calculated the theoretical semantic centrality (*Cen(j)*) of every word by averaging their relative connection strength with all other words since all words could serve as their associated cue.

#### Memory performance explanation analyses

To explore whether semantic centrality and other target variables can explain observed word memory performance, we analyzed data using a generalized linear mixed model (GLMM) approach^49,50^ as implemented by the *lme4* package^51^ in R (https://www.r-project.org). Since we were concerned with a binary dependent variable (DV), namely successful vs. unsuccessful recall on each trial, a binominal distribution and logistic link function were used for model fitting. The output from each individual trial of the cued recall task from every participant was modelled in two ways to interrogate the data fully: (1) a single explanatory variable (EV) approach, which incorporated a single target word property as a fixed effect, and target stimulus and participant ID as random effects; and (2) a full EV approach, which incorporated all target word properties as fixed effects, and target and participant ID as random effects. The target word properties that were included in these models were semantic centrality, frequency (log-transformed), concreteness, valence, arousal, age of acquisition, number of character strokes, and target position. Target position coded for the original encoding spatial positions of the cue and target words, and allowed us to consider the possibility that performance would be worse if the cue and target encoding positions violated the direction in which Chinese characters are usually read (i.e., from left to right). If the cue was on the right of the target during encoding, the variable was coded as 1, otherwise coded as 0.

All models were initially specified with a maximal random effects structure^52^, which included the by-subject random slope of the EV, by-subject random intercept, and by-target random intercept:

$$\:DV\:\sim\:1+EV+\left(1+EV\:\right|\:subject\:ID)+\left(1\:\right|\:target\:ID)$$

While the by-subject random intercept and slope capture participant differences in performance and the impact of the EVs, the by-target random intercept captures inherent differences in the memorability of the words (i.e. stable item-level propensity for certain stimuli to be remembered more reliably than others across observers). In other words, word memorability is always accounted for in the GLMM, and word-level properties such as semantic centrality are then included as fixed effects to examine whether they explain additional systematic variance in memory performance. Note that since there was only one level for each fixed effect, a by-target random slope was not necessary. Before fitting models, all continuous variables were rescaled into z scores. If the maximal model failed to converge, components of the maximal random structure were gradually removed until convergence was achieved.

To assess the fit of a single EV model, an identical model was fit to the data but with the fixed effect removed. A likelihood-ratio statistical test was then conducted between these two models to quantify whether the model with the fixed effect was a better fit.

To assess the fit of the full EV model, a backward stepwise selection of fixed effects was implemented using the *GLMERSelect* function from an open-source R package (rdrr.io/github/timnewbold/StatisticalModels/man/GLMERSelect.html). This produced the optimal parsimonious model of explaining memory performance with a minimal number of fixed effects, while including static random effects, i.e., by-stimulus and by-subject random intercepts.

Although we were primarily interested in how task performance was influenced by target word properties, in line with Xie et al.^9^, we also explored the contributions of cue word properties by running the same single EV models but with cue word properties and by-cue random intercepts instead of target word properties and by-target random intercepts.

#### Intrusion error analysis

We also explored whether semantic centrality can account for intrusion errors, specifically, the probability that a previously encoded word was recalled incorrectly in response to a cue word. We only included participants with at least ten intrusion error trials, in keeping with Xie et al.^9^ We calculated the mean semantic centrality of these intrusion errors for each participant and conducted a one-sample t-test to examine whether this mean value was significantly different from the median semantic centrality value of all words.

### Results

**Fig. 3:**
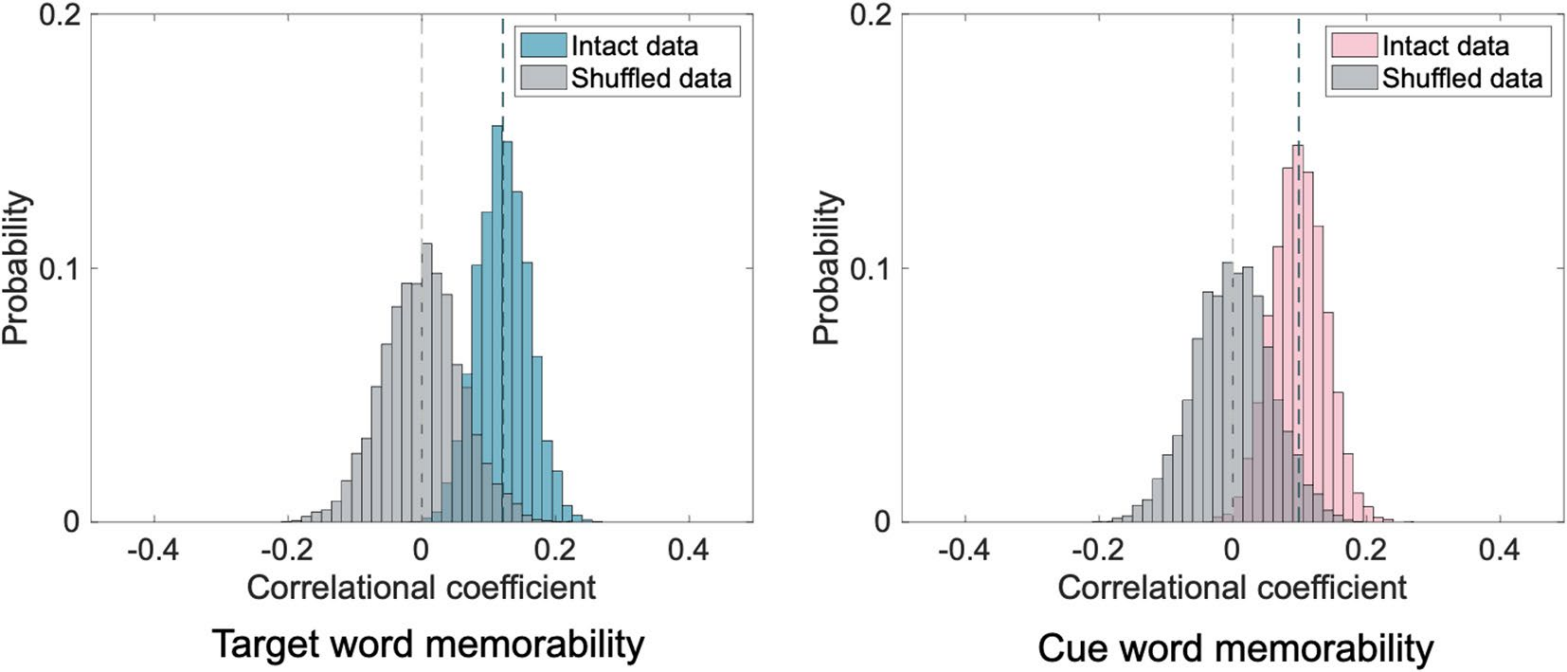
The probability density distribution of correlation coefficients between word memorability calculated from two random halves of participants.

The mean accuracy of all participants on the cued recall task was 82.18% (SD = 11.91%). The random split-half analysis (5,000 iterations) revealed consistent word memorability observed across participants, with a Spearman rho 95% CI of [0.047, 0.195] (Fig. 3). In comparison, the Spearman rho 95% CI when the word labels in one split-half group were shuffled was [-0.115, 0.117]. For cues, there was also consistent memorability across participants (Spearman rho 95% CI [0.022, 0.177], shuffled Spearman rho 95% CI [-0.116, 0.114]).

The single EV GLMMs revealed significant effects of semantic centrality (*p* < .001), the number of character strokes (*p* = .026), and target position (*p* = .035), with all other target word properties having no significant impact on memory performance (see Table 1 for full results). Surprisingly, the lower the semantic centrality, the higher the probability of successful cued recall ($\:\widehat{{\upbeta\:}}$ = -0.125). This contrasts with the findings of Xie et al.^9^ with English words and raises the possibility that in Chinese, the relationship between semantic centrality and memory performance is non-linear, with words with medium semantic centrality being the most memorable. However, when we considered an alternative model with a quadratic term of semantic centrality added, it did not show a better model fit in contrast to the original one (*p* = .496). On the other hand, characters with a higher number of strokes ($\:\widehat{{\upbeta\:}}$ = -0.082) and targets that were learned on the left ($\:\widehat{{\upbeta\:}}$ = -0.163) were both less likely to be recalled successfully. We additionally explored the contribution of cue properties to memory performance (see Table S2, S4). Notably, cue semantic centrality also significantly predicted retrieval success in the negative direction ($\:\widehat{{\upbeta\:}}$ = -0.158, *p* < .001).

**Table 1:** Single EV GLMM results for experiment 1.

EV	Random effects	χ^2^	df	*p*	$\:\widehat{{\upbeta\:}}$	SE
Semantic centrality	(1|subID) + (1|tarID)	11.268***	1	< 0.001	– 0.125	0.037
Concreteness	(0 + EV|subID) + (1|subID) + (1|tarID)	0.017	1	0.897	0.005	0.037
Frequency	(1|subID) + (1|tarID)	1.272	1	0.260	0.042	0.037
Valence	(1 + EV|subID) + (1|tarID)	2.206	1	0.138	0.061	0.040
Arousal	(1 + EV|subID) + (1|tarID)	0.670	1	0.413	0.037	0.044
Age of acquisition	(1|subID) + (1|tarID)	0.023	1	0.881	– 0.006	0.037
Number of strokes	(1|subID) + (1|tarID)	4.956*	1	0.026	– 0.082	0.036
Target position	(1 + EV|subID) + (1|tarID)	4.428*	1	0.035	– 0.163	0.074

*: *p* < .05; **: *p* < .01; ***: *p* < .001. subID: subjectID, tarID: targetID.

For the full EV model, a backward stepwise selection from all EVs led to two fixed effects remaining, specifically semantic centrality ($\:\widehat{{\upbeta\:}}$ = -0.125, *p* < .001) and target position ($\:\widehat{{\upbeta\:}}$ = -0.186, *p* = .003).

Finally, intrusion errors for words that had been previously presented in the same or an earlier encoding-recall block had a significantly higher semantic centrality than the median value (independent sample t-test, *t*(21) = 2.42, *p* = .025).

### Discussion

In this experiment of Chinese two-character neutral nouns, we found reliable word memorability that was explained by semantic centrality as well as the number of character strokes and the position of the target character during encoding. The directionality of the latter two was as expected: a higher number of strokes and a target encoded on the left were associated with decreased memory performance in the cued-recall task. Surprisingly, however, lower semantic centrality corresponded to a higher probability of successful recall, which suggests that words that were less semantically connected were more memorable and is the opposite of what was reported by Xie et al.^9^ Notably, our full EV model isolated semantic centrality and target position as significant fixed effects, suggesting, therefore, that these factors account for unique variance in memory performance.

Having identified some of the semantic factors that may influence the memorability of neutral Chinese words, we next sought to examine the potential influence of emotion. To this end, we conducted Experiments 2 and 3, with the latter being a replication of the former, using a word pool of positive, negative, and neutral nouns.

## Experiments 2 & 3

### Methods

#### Participants

Fifty-nine and sixty participants (Experiment 2: 28 females, mean age = 21.46, SD = 3.14 years; Experiment 3: 33 females, mean age = 21.97, SD = 3.13 years) were recruited in Experiments 2 and 3 respectively, from the Tsinghua University student population and received monetary compensation for their time. All participants had Chinese as their singular first language and self-reported to have no historical or current mental and/or neurological disorders. Prior to the experiment, participants signed an informed consent form. The study was approved by the local Ethics Committee at Tsinghua University. All procedures were performed in accordance with the relevant guidelines and regulations and with the Declaration of Helsinki. One male participant in Experiment 2 misunderstood the task instructions and did not type the target word during the cued recall phase and hence, their data were excluded. All other participants’ data were included in the final analyses (Experiment 2: 28 females, mean age = 21.47, SD = 3.17 years; Experiment 3: 33 females, mean age = 21.97, SD = 3.13 years). One participant’s data in Experiment 3 only contained ten instead of thirteen blocks due to a computer malfunction. Data, analysis codes, and stimuli for all experiments are available at osf.io/7bhgk/.

#### Materials

From the MELD-SCH database used in Experiment 1, we selected another set of 100 positive (mean valence = 5.47, SD = 0.36), 100 negative (mean valence = 2.23, SD = 0.47), and 100 neutral (mean valence = 4.27, SD = 0.45) two-character nouns. All words were manually checked to ensure that they had direct unambiguous translations in English. None of the words used in the experiment had the same PinYin, although among all possible word pairs, 0.6% of them shared a common character. Within the context of a 7-point Likert scale, words with a valence rating larger than 5 and smaller than 3 were categorized as positive and negative words respectively, and the remaining words were regarded as neutral words. All nouns were moderate to high frequency (log-transformed frequency in the database mean = 2.87, SD = 0.37, min = 2.02, max = 3.89), and were matched across valence categories with respect to frequency, concreteness, arousal, age of acquisition, and number of strokes (*ps* > 0.25, see Table 2). As expected, there was a significant difference in valence across word groups (*p* < .001) with post-hoc tests (Tukey-Kramer corrected) revealing a significant difference for all pairwise comparisons (all *p*s < .001). There was also a significant difference between the three groups with respect to semantic centrality (*p* < .001). Post-hoc comparisons (Tukey-Kramer corrected) revealed a significantly higher semantic centrality for negative compared to positive words (*p* = .002) and neutral words (*p* < .001), while positive and neutral words’ semantic centrality did not differ significantly (*p* = .822). Notably, the range of the words’ concreteness ratings (mean = 3.51 out of 5, SD = 0.76, min = 1.48, max = 4.85) was wider than that in Experiment 1, which was unavoidable since many positive and negative words tend to be relatively low in concreteness (e.g., *gift* and *noise*). Nevertheless, we restricted our stimulus selection to the upper end of the concreteness distribution while preserving an ecologically valid set of emotionally valenced words. Finally, a technical error unfortunately led to 3 neutral nouns being picked twice in Experiment 2 leading to a single trial in which two identical nouns were presented. Data from this trial were excluded and this error was corrected in Experiment 3. Since we used all the words in the pool as stimuli once in the experiment, all participants saw exactly 100 positive, negative, and neutral words in total. As mentioned in Experiment 1, given that there were 44,850 possible word pairs, each participant encountered a unique set of word pairs.

**Table 2:** One-way ANOVA of word properties among emotion groups.

Word property	F	*p*	Positive group		Negative group		Neutral group	
			Mean	SD	Mean	SD	Mean	SD
Valence	1452.03	< 0.001	5.47	0.36	2.23	0.47	4.27	0.45
Semantic centrality^1^	9.15	< 0.001	– 0.13	0.98	0.34	0.95	– 0.21	0.98
Concreteness	0.54	0.58	3.45	0.82	3.56	0.72	3.51	0.75
Frequency	0.70	0.50	2.89	0.39	2.84	0.34	2.89	0.39
Arousal	1.31	0.27	2.63	0.40	2.71	0.43	2.62	0.36
Age of acquisition	0.82	0.44	10.60	2.38	10.99	2.02	10.85	2.18
Number of strokes	1.38	0.25	16.07	4.19	16.98	3.87	16.48	3.48

^1^z scores for semantic centrality means and SDs. All other means and SDs are from raw scores in the database.

#### Procedure

Experiments 2 and 3 were identical in procedure to Experiment 1 with the exception that in addition to neutral words, emotional word stimuli were used. Word pairs were selected randomly without considering their emotions, and hence all possible pairings of emotion were presented (e.g., positive-negative, positive-positive, negative-negative, positive-neutral, etc.).

### Data analysis

**Fig. 4:**
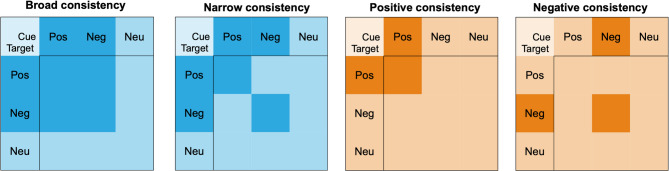
Depiction of the four different types of emotional consistency. Pos = Positive; Neg = Negative; Neu = Neutral.

We implemented the same analyses as in Experiment 1 but additionally specified the emotional consistency between the cue and target words on each trial as an EV in our models. This was done in one of four ways (Fig. 4) using binary coding:

1. Broad consistency – here, we considered instances where both the cue and target words were emotional, regardless of valence. Thus, all trials with positive-positive, negative-negative, or positive-negative word pairs were coded as 1 and all other trials as 0.
2. Narrow consistency – here, we considered cases where both the cue and target words were of the same valence. Thus, all trials with positive-positive or negative-negative word pairs were coded as 1 and all other trials as 0.
3. Positive consistency – only trials with positive-positive word pairs were coded as 1 and all other trials as 0.
4. Negative consistency – finally, we coded negative-negative word pair trials as 1 and all other trials as 0.

Broad emotional consistency allowed us to assess whether a general form of emotional consistency (i.e., regardless of valence direction) results in higher memory performance. For comparison, we also included narrow emotional consistency (same-valence pairs) as an alternative theoretical account, based on the proposal that valence alignment may facilitate integrative encoding rather than emotional salience alone (i.e., broad consistency). Finally, positive and negative emotional consistency were included primarily for comparison with prior studies^30,33^, which reported significant associative memory enhancement for positive-positive word pairings. The results of positive consistency were expected to replicate the previously found memory enhancement patterns, while negative consistency served as a control model in the analysis. Notably, the emotional consistency variables captured the relationship between the cue and target rather than item-level emotional properties. This modeling choice is theory-driven and aligns with prior findings showing that emotionally congruent pairs (e.g., positive-positive) are remembered better than mixed-valence pairs (e.g., positive-neutral ^30)^. For comparison, we also fit GLMMs that included cue-valence category, target-valence category, and their interaction as fixed effects (see Table S5). These analyses did not reveal any consistent pattern of EV effects across the two experiments.

To preview our findings, single EV GLMMs revealed a significant effect of semantic centrality, broad emotional consistency, and positive emotional consistency (see Table 4). We, therefore, also explored the additive effects of these factors and their potential interaction on memory performance. Specifically, we used the backward stepwise procedure (see Experiment 1 Methods) with two follow-up dual-EV GLMMs with fixed effects of either: (1) semantic centrality, broad emotional consistency and their interaction; or (2) semantic centrality, positive emotional consistency, and their interaction.

Finally, we conducted full-EV GLMMs by re-running these GLMM models (i.e., with semantic centrality, emotional consistency, and their interaction as fixed effects) with all other target properties incorporated in the stepwise selection procedure. One theoretical motivation for the full-EV GLMMs was to test whether emotional consistency continues to predict associative memory when other lexical-semantic properties (e.g., concreteness) are considered. This question relates to the broader inquiry on whether emotional effects on memory can be explained by semantic factors^36,37^.

To investigate the relationship between semantic centrality and intrusion errors, similar analyses to that in Experiment 1 were run. We hypothesized that intrusion errors were likely to be emotionally consistent with the presented cue and posited one specific hypothesis for each type of emotional consistency. For broad emotional consistency, we hypothesized that among intrusion error trials with non-neutral cues, the proportion of non-neutral intrusion errors would be higher than chance level (66.67%). For narrow emotional consistency, we hypothesized that among intrusion error trials with non-neutral cues, the proportion of intrusion errors with the same valence (positive or negative) as the cue would be higher than chance level (33.33%). Finally, for positive/negative emotional consistency, we hypothesized that among intrusion error trials with positive/negative cues, the proportion of positive/negative intrusion errors would be also higher than the chance level (33.33%). For each hypothesis, we conducted a one-sample t-test and included only participants with at least ten trials of interest.

### Results

**Fig. 5:**
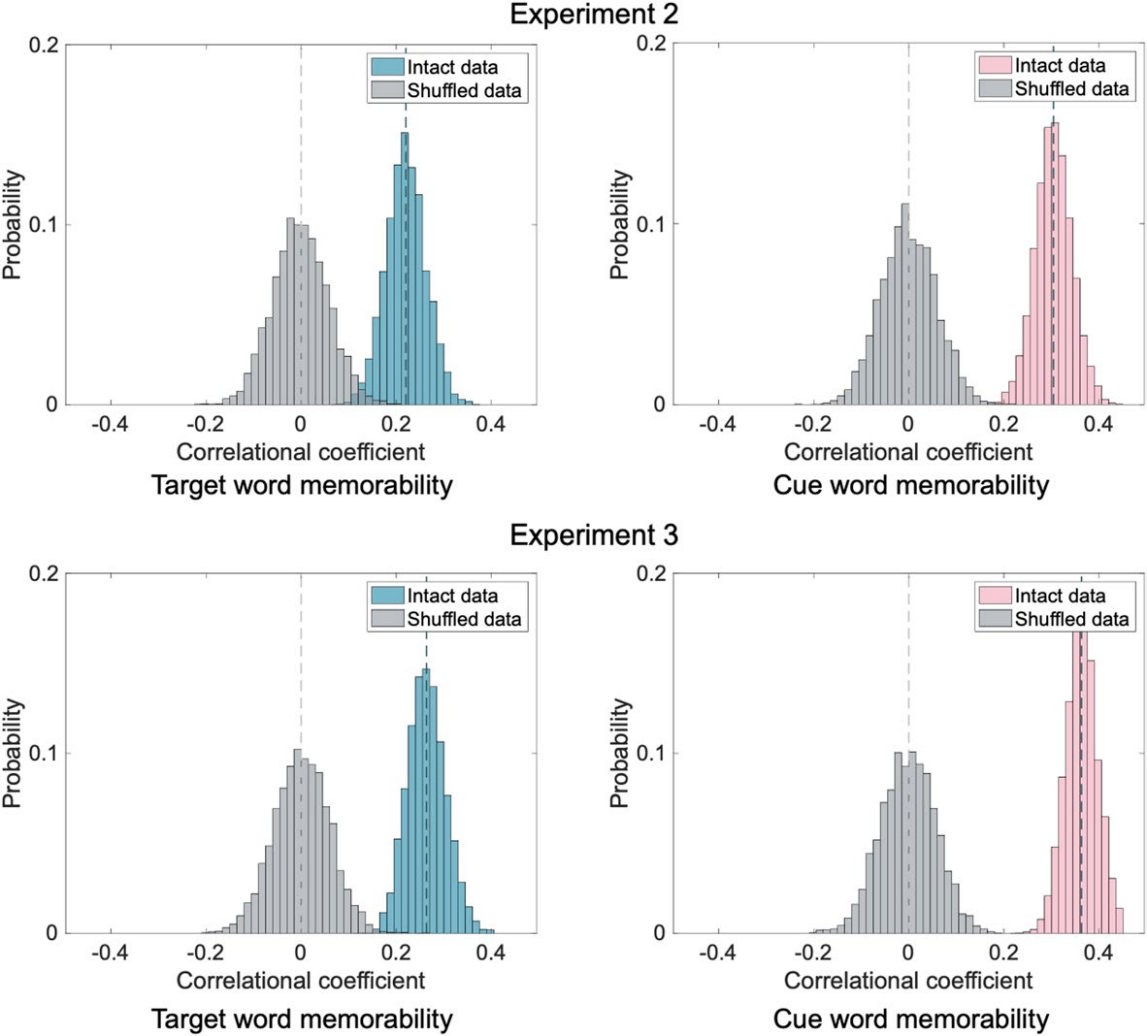
The probability density distributions of correlation coefficients between word memorability calculated from two random halves of participants.

Mean participant accuracy was 67.64% (SD = 20.26%) in Experiment 2 and 63.67% (SD = 17.97%) in Experiment 3. For both experiments, consistent target word memorability was observed across participants (Experiment 2 Spearman rho 95% CI: [0.137, 0.294]; Experiment 3 Spearman rho 95% CI: [0.186, 0.332]; Fig. 5). In comparison, the Spearman rho 95% CIs when the word labels in one split-half group were shuffled were [-0.114, 0.112] in Experiment 2 and [-0.115, 0.114] in Experiment 3. Cues also showed consistent memorability across participants in both experiments (Experiment 2 Spearman rho 95% CI: [0.227, 0.380]; Experiment 3 Spearman rho 95% CI: [0.297, 0.430]), while the shuffled data did not (Experiment 2 Spearman rho 95% CI: [-0.112, 0.114]; Experiment 3 Spearman rho 95% CI: [-0.113, 0.110]).

**Fig. 6:**
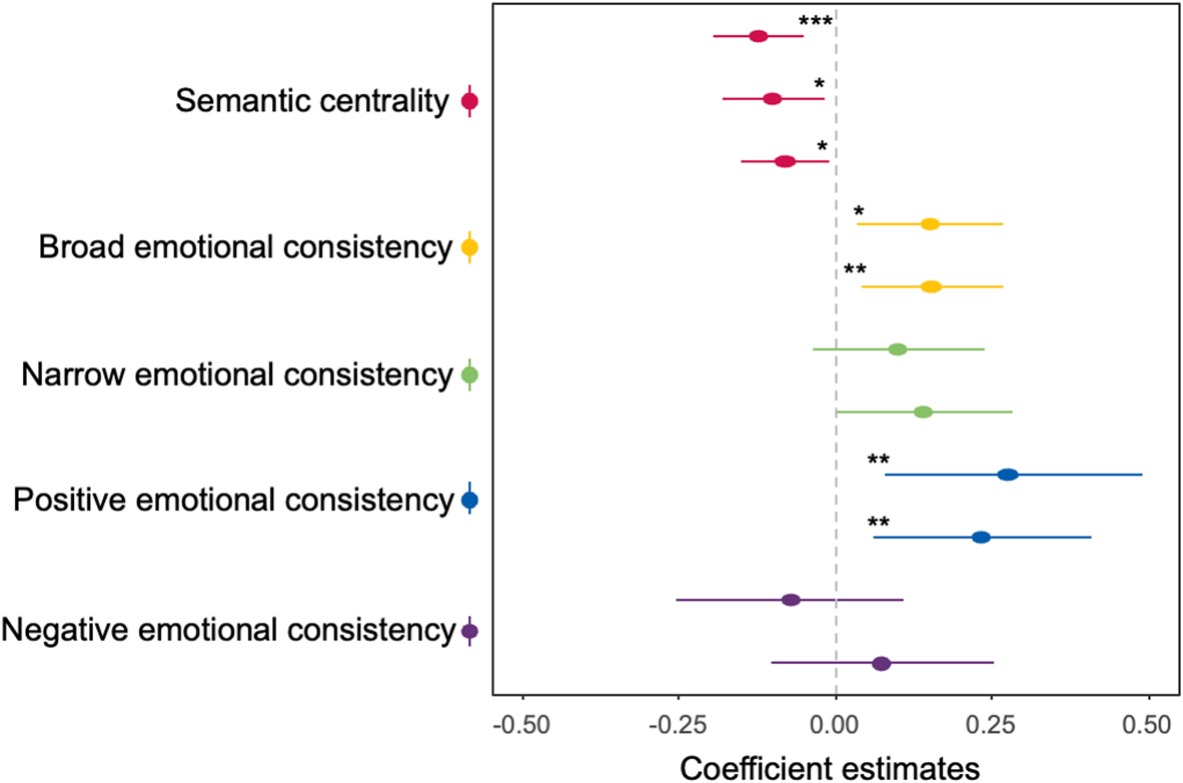
Single EV GLMM coefficient estimates and their respective 95% confidence intervals for semantic centrality (red lines top to bottom: Experiments 1, 2, and 3) and the four different types of emotional consistency (yellow, green, blue, and purple lines top: Experiment 2, bottom: Experiment 3). The significance labels (*: *p* < .05; **: *p* < .01; ***: *p* < .001) represent the *p*-value from the model comparison likelihood-ratio test.

Similar to Experiment 1, single EV GLMMs (Fig. 6) revealed a significant effect of semantic centrality in both Experiment 2 (*p* = .017) and Experiment 3 (*p* = .021), with a significant negative relationship between semantic centrality and the probability of successful cued recall (Experiment 2: $\:\widehat{{\upbeta\:}}$ = -0.100; Experiment 3: $\:\widehat{{\upbeta\:}}$ = -0.081; see Table 4 for full results). Adding a quadratic term of semantic centrality to the model did not provide a better model fit for either experiment (Experiment 2: *p* = .814; Experiment 3: *p* = .247). Similarly, cue semantic centrality significantly and negatively predicted memory performance (Experiment 2: $\:\widehat{{\upbeta\:}}$ = -0.227, *p* < .001; Experiment 3: $\:\widehat{{\upbeta\:}}$ = -0.194, *p* < .001; see Table S3-S4 for full summaries of cue property results).

With regards to the impact of emotion, broad emotional consistency explained memory performance in both experiments (Experiment 2: *p* = .013; Experiment 3: *p* = .008) with word pairs consisting of two non-neutral words associated with a higher possibility of cued-recall (Experiment 2: $\:\widehat{{\upbeta\:}}$ = 0.150; Experiment 3: $\:\widehat{{\upbeta\:}}$ = 0.153). In contrast, narrow emotional consistency failed to explain word memory in either experiment (Experiment 2: *p* = .151; Experiment 3: *p* = .051). Finally, positive consistency was also able to account for word memory (Experiment 2: *p* = .008; Experiment 3: *p* = .009) with a positive relationship between the two (Experiment 2: $\:\widehat{{\upbeta\:}}$ = 0.275, *SE* = 0.105; Experiment 3: $\:\widehat{{\upbeta\:}}$ = 0.233, *SE* = 0.089) whereas negative consistency failed to explain behavioural performance (Experiment 2: *p* = .434; Experiment 3: *p* = .425).

Single EV GLMMs also revealed that concreteness was positively related to cued recall success in Experiment 2 (*p* < .001, $\:\widehat{{\upbeta\:}}$ = 0.160) and Experiment 3 (*p* < .001, $\:\widehat{{\upbeta\:}}$ = 0.186). Furthermore, there was a significant positive relationship between target word frequency and cued recall performance in Experiment 3 (*p* = .006) but not in Experiment 2 (*p* = .207), with no other target word properties reaching significance (Table 3, also Table 4 for random effect structure).

**Table 3:** Single EV GLMM Results. For χ^2^, *p*, $\:\widehat{{\upbeta\:}}$, SE, left value = Experiment 2, right value = Experiment 3.

EV	χ^2^	df	*p*	$\:\widehat{{\upbeta\:}}$	SE
Semantic centrality	5.723*; 5.289*	1	0.017; 0.021	– 0.100; – 0.081	0.040; 0.035
EmoConsist: Broad	6.174*; 6.977**	1	0.013; 0.008	0.150; 0.153	0.060; 0.058
EmoConsist: Narrow	2.066; 3.813	1	0.151; 0.051	0.100; 0.141	0.069; 0.071
EmoConsist: Positive	7.022**; 6.846**	1	0.008; 0.009	0.275; 0.233	0.105; 0.089
EmoConsist: Negative	0.613; 0.644	1	0.433; 0.422	– 0.073; 0.071	0.092; 0.089
Concreteness	14.809***; 22.106***	1	< 0.001; <0.001	0.160; 0.186	0.038; 0.036
Frequency	1.593; 7.507**	1	0.207; 0.006	0.050; 0.097	0.040; 0.035
Valence	2.384; 0.051	1	0.123; 0.822	0.053; 0.008	0.034; 0.035
Arousal	1.150; 1.690	1	0.284; 0.194	0.037; 0.048	0.034; 0.037
Age of acquisition	0.260; 0.650	1	0.610; 0.420	– 0.018; – 0.031	0.035; 0.038
Number of strokes	0.991; 2.490	1	0.320; 0.115	– 0.034; – 0.056	0.034; 0.035
Target on the left	1.407; 1,547	1	0.236; 0.214	– 0.066; – 0.065	0.055; 0.052

*: *p* < .05; **: *p* < .01; ***: *p* < .001; EmoConsist: Emotional Consistency.

Given the significant effects of semantic centrality, broad emotional consistency, and positive emotional consistency, we further explored possible interactions between semantic centrality and each of these two types of emotional consistency using a stepwise selection procedure for dual EV models. The additive model was consistently selected as the final model with the best explanation power, with the added specification of an interaction between semantic centrality and emotional consistency failing to provide a significantly better fit in either study, for broad emotional consistency (Experiment 2: χ^2^(1) = 0.201, *p* = .654; Experiment 3: χ^2^(1) = 1.749, *p* = .186) or positive emotional consistency (Experiment 2: χ^2^(1) = 0.188, *p* = .665; Experiment 3: χ^2^(1) = 3.046, *p* = .081). The direction of relationship between semantic centrality/emotional consistency and recall probability remained unchanged (i.e., a negative relationship between semantic centrality and recall probability, and a positive relationship between broad/positive emotional consistency and recall probability).

**Table 4:** Single EV GLMM random effect structure.

Variable	Experiment 2	Experiment 3
Semantic centrality	(1 + EV|subID) + (1|tarID)	(1|subID) + (1|tarID)
EmoConsist: Broad	(1|subID) + (1|tarID)	(1|subID) + (1|tarID)
EmoConsist: Narrow	(1|subID) + (1|tarID)	(1 + EV|subID) + (1|tarID)
EmoConsist: Positive	(1 + EV|subID) + (1|tarID)	(1|subID) + (1|tarID)
EmoConsist: Negative	(1|subID) + (1|tarID)	(1|subID) + (1|tarID)
Concreteness	(1 + EV|subID) + (1|tarID)	(1 + EV|subID) + (1|tarID)
Frequency	(1 + EV|subID) + (1|tarID)	(1|subID) + (1|tarID)
Arousal	(1|subID) + (1|tarID)	(1 + EV|subID) + (1|tarID)
Age of acquisition	(0 + EV|subID) + (1|subID) + (1|tarID)	(1 + EV|subID) + (1|tarID)
Number of strokes	(1|subID) + (1|tarID)	(1|subID) + (1|tarID)
Target on the left	(1|subID) + (1|tarID)	(1|subID) + (1|tarID)

EmoConsist: Emotional Consistency; subID: subjectID, tarID: targetID.

For a full EV model incorporating broad emotional consistency, backward stepwise selection resulted in final fixed effects of broad emotional consistency, semantic centrality, and concreteness in Experiment 2 and broad emotional consistency, frequency, and concreteness in Experiment 3. Similar results were observed for a full EV model incorporating positive emotional consistency. In Experiment 2, the final model included emotional consistency and concreteness as fixed effects, while in Experiment 3, the model’s fixed effects were emotional consistency, frequency, and concreteness. Table 5 summarizes the findings of the dual EV and full EV model analyses for all three experiments. In summary, both broad and positive emotional consistencies were found to explain unique variance when considering all other target properties, while semantic centrality dropped out in three out of five full-EV final models.

**Table 5:** Dual and full EV model fixed effects for experiments 1, 2, and 3.

Experiment	Emotional consistency	Dual EV fixed effects	Full EV fixed effects
Experiment 1	N/A	N/A	Semantic centrality and target position
Experiment 2	Broad	Emotional consistency and semantic centrality	Emotional consistency, semantic centrality, and concreteness
	Positive	Emotional consistency and semantic centrality	Emotional consistency and concreteness
Experiment 3	Broad	Emotional consistency and semantic centrality	Emotional consistency, frequency, and concreteness
	Positive	Emotional consistency and semantic centrality	Emotional consistency, frequency, and concreteness

Since broad/positive emotional consistency and concreteness remained in all optimal models in the full EV analyses, we compared models with and without an interaction term. Results showed that there was no evidence for an interaction between target concreteness and either type of emotional consistency (broad emotional consistency: *ps* = 0.095 and 0.571; positive emotional consistency: *ps* = 0.883 and 0.532 in Experiments 2 and 3 respectively).

To aid the interpretation of the emotional consistency results, the grand mean frequency of successful recall for every combination of cue-target emotion is summarized in Table 6. The average memory performance of mixed emotion pairs, especially cue-negative target-positive pairs, was similar to that for positive-positive pairs and relatively high compared to other conditions. This suggests that the observed enhanced memory for broad emotional consistency pairs is not driven solely by positive-positive word pairs. Furthermore, we conducted a supplementary GLMM analysis using the three-level valence categories (negative, neutral, positive) for cues and targets, including their main effects and interactions as fixed effects. Using two orthogonal contrasts — the *salient* contrast (Negative = 1, Neutral = -2, Positive = 1) and the *positive* contrast (Negative = -1, Neutral = 0, Positive = 1) — we found that both general emotional salience and positive valence contributed to cued-recall performance, although the specific predictors differed between Experiments 2 and 3 (Table S5).

**Table 6:** Average recall success frequencies for all emotion combinations in experiments 2 and 3.

Experiment 2				Experiment 3			
Target \ Cue	Positive	Negative	Neutral	Target \ Cue	Positive	Negative	Neutral
Positive	71.4%	67.8%	67.6%	Positive	67.7%	62.9%	65.1%
Negative	71.2%	66.4%	62.1%	Negative	66.2%	64.9%	59.1%
Neutral	68.8%	65.9%	67.5%	Neutral	60.7%	64.7%	61.3%

Finally, the semantic centrality of intrusion errors for words that were previously presented in the same or an earlier encoding-recall block was higher than the median semantic centrality value in both experiments (Experiment 2: *t*(28) = 5.12, *p* < .001; Experiment 3: *t*(35) = 5.94, *p* < .001). Moreover, in both experiments narrow emotional consistency (Experiment 2: *t*(15) = 4.24, *p* < .001; Experiment 3: *t*(27) = 2.09, *p* = .046) and positive emotional consistency (Experiment 2: *t*(3) = 3.19, *p* = .050; Experiment 3: *t*(8) = 2.55, *p* = .034) significantly explained the valence of intrusion errors. Significant effects were not observed for broad emotional consistency (Experiment 2: *t*(15) = 2.04, *p* = .059; Experiment 3: *t*(27) = 1.51, *p* = .142) or negative emotional consistency (Experiment 2: *t*(6) = 2.09, *p* = .081; Experiment 3: *t*(12) = 1.28, *p* = .226).

### Discussion

In Experiments 2 and 3, we used a stimulus pool of positive, negative, and neutral nouns to investigate the impact of emotional consistency on word memory. We found that certain types of emotional consistency, namely broad and positive consistency, were associated with enhanced cued-recall success. These two consistency definitions are different but compatible, reflecting a broad consistency effect among non-neutral pairs, with positive pairs possessing the strongest connection and hence highest memory performance. Similar to Experiment 1, there was a negative relationship between semantic centrality and memory performance, with the former showing an additive but not interactive effect with both emotional consistencies. Of note, however, while the impact of emotional consistency remained significant even when all other target properties were considered, the effect of semantic centrality was less consistent, suggesting the relatively weak impact of semantic centrality (when compared to factors such as emotional consistency and concreteness) on target word recall.

## General discussion

We examined whether semantic centrality and emotional consistency can account for Chinese word memorability in the context of associative memory. Surprisingly but consistently across all three experiments, we found that words with weaker semantic connections were more memorable, a finding that is contrary to Xie et al.^9^, which reported that greater semantic centrality is associated with increased memorability. Moreover, we observed that broad emotional word pairs (i.e., non-neutral pairs) were more memorable than other pairs, with positive-positive pairs having the highest memory performance. Finally, there was a stable additive but not interactive effect between semantic centrality and broad emotional consistency, as well as between semantic centrality and positive emotional consistency.

Although the observed direction of the relationship between semantic centrality and memory performance is puzzling when considering Xie et al.^9^, it is not necessarily illogical. Specifically, words that are less semantically related may be less indistinct and hence, recalled more easily in a cued recall paradigm. Indeed, semantic centrality may be disruptive during memory retrieval, as observed in the Deese/Roediger/McDermott paradigm^53–56^. One intriguing question for further investigation is whether this finding is specific to the type of words used in the current study (i.e., commonly used nouns) or whether it extends to other word categories (e.g., adjectives or uncommon professional jargon). A potential factor underlying the observed effects is unitization, the degree to which word pairs can be encoded as a single, integrated representation^57–59^. Prior work has shown that unitized pairs (e.g., traffic-jam) can be better remembered than semantically similar but weakly unitizable pairs (e.g., lemon-orange)^59^. One possibility, therefore, is that certain semantically connected word pairs are less amenable to unitization, as their conceptual overlap hinders the formation of a unified representation. However, our focus on target memorability across different cues makes it unlikely for the observed effects to be solely driven by cue-target unitization. It remains an open question whether unitization plays a stronger role in Chinese than in English, potentially due to the productive compounding structure of Chinese. Future work could more directly examine how semantic connection and unitization jointly shape associative memory.

The discrepancy between the current work and Xie et al.^9^ may be explained, at least in part, by the many differences between the Chinese and English languages. For example, the use of logographic characters in Chinese, as opposed to a Latin alphabet writing system in English, may be associated with stronger visual encoding/cognitive processing^60–62^. Despite our efforts to minimize language effects by using common nouns with direct unambiguous translations, our results may reflect subtle differences in the semantic mechanisms/structure of Chinese and English, which require additional insights from linguistics and further empirical work. Speculating further, the observed discrepancy could also be attributed to other cultural differences between mainland Chinese and North American participants, in particular with regard to holistic versus analytic cognition theory^24,63,64^. Specifically, this long-standing theory in cultural psychology categorizes East Asians as holistic thinkers, with a propensity to relate objects to their context when explaining and predicting events, while North Americans are categorized as analytical thinkers, decontextualizing objects and focusing on their attributes alone to explain and predict events. It is possible that a tendency to think holistically results in words with stronger semantic connections becoming less distinctive and therefore, less memorable. Conversely, in analytic thinking cultures, strong semantic relationships between words are essential for memory retention, leading to the opposite relationship between semantic centrality and word memory performance. Previous research has shown that holistic/analytical thinking can account for performance differences in various cognitive tasks^65,66^. For instance, presenting words with an unrelated background image facilitated later word recall for Chinese but not for American participants, which was interpreted as a “binding” effect specific to holistic thinkers^24,67^. Future studies are needed to examine how the relationship between semantic centrality and word memory may vary across languages and cultural contexts. A valuable extension of the present work will be to incorporate bilingual samples and within-subject cross-language stimulus designs to further dissociate language-specific effects from population-related influences. In addition, incorporating individual-level cultural measures (e.g., Analysis-Holism Scale^68)^ could facilitate evaluating the modulatory role of cultural cognitive factors (e.g., analytic versus holistic thinking) in semantic centrality effects on associative memory. Finally, a direct comparison across languages will allow the relative contribution of cue versus target properties to memory performance to be examined.

We highlight that there was a notable difference in performance accuracy between the current participants (Expt 1: 82.2% on average; Expt 2: 67.6%; Expt 3: 63.7%) and those in Xie et al.^9^ (30.2% and 51.7%). This difference in accuracy is likely to be driven by participant population differences between the two studies, with university students having been assessed in the present study, and patients with epilepsy and online participants from a crowdsourcing marketplace (i.e., Amazon MTurk) making up the participant pool in Xie et al.^9^ Notably, however, previous work has demonstrated that while population characteristics can influence absolute values of performance accuracy, they do not significantly impact the rank order of stimulus memorability in young children^96^ or epileptic patients^9^. Thus, it is unlikely that differences in cognitive ability between participant groups can account entirely for the observed difference in the relationship between semantic centrality and word memory in the current study and Xie et al.^9^ Moreover, it might be that our word embedding model and the one used in Xie et al.^9^, GloVe^69^, are heterogenous and therefore predict word memory in opposing directions. However, a post hoc analysis indicates this is not the case (see Supplementary Result S1): semantic centrality estimates using the two embeddings (i.e., Chinese word2vec embeddings and translated English GloVe embeddings) are positively correlated for both the neutral and emotional word sets in our study.

Regarding emotional consistency, both Experiments 2 and 3 highlighted broad and positive emotional consistency, but not narrow or negative emotional consistency, as factors contributing to memory performance. The observation that positive-positive word pairs are associated with better word memory compared to other pair types converges with previous studies^30,33^. The strengthened connection between positive targets and cues aligns with theoretical accounts positing that positive information facilitates associative binding. Related supporting evidence comes from broader memory research (i.e., autobiographical memory) suggesting that individuals tend to remember positive experiences more holistically^70,71^, although important differences remain between laboratory word-memory tasks and everyday autobiographical recall. This memory effect may potentially underpin the role of positive emotion in flexible and creative thinking^72,73^, by retaining the focus on item connections that can be created and manipulated flexibly^74^. One open question for future work is whether words belonging to the same fine-grained category of positive emotion (e.g., joy and happiness) are more memorable in the context of an associative memory paradigm compared to words belonging to different categories of positive emotions^75,76^.

It is also worth noting that overall, non-neutral pairs were more memorable than pairs containing at least one neutral item. This suggests that in associative memory, the pairing of two emotional words confers a memory advantage, regardless of their valence and even when their valence is non-identical. The latter aligns with research suggesting that the processing and remembering of mixed emotions (i.e., a mixture of positive and negative feelings) can be prioritized^77–79^. Moreover, our finding of a valence-related memory advantage should be considered alongside reports of null valence effects in word recall^36,80^. These discrepancies likely reflect differences in task demands, encoding processes, and stimulus control. Unlike free or serial recall tasks used in prior studies, our associative cued-recall paradigm required cue-target binding. This design may have promoted relational encoding, whereas null effects are more commonly observed in tasks emphasizing item encoding processing (e.g., rehearsal). In addition, we carefully controlled for lexical and semantic variables (e.g., concreteness, frequency), reducing variance unrelated to emotion and allowing valence effects to emerge. Thus, emotional facilitation in memory may not necessarily be absent but rather depend on task structure and processing demands.

We also incorporated several target semantic and emotional properties into our analyses, which yielded a number of intriguing findings. Consistent with Xie et al.^9^, target concreteness failed to explain word memory in Experiment 1 although it is important to note that the words used in both these cases were drawn from a restricted range of concreteness, specifically high concreteness. In contrast, when Experiments 2 and 3 used words with moderate to high concreteness, concreteness turned out to be the strongest predictor of word memorability among all variables, consistent with previous findings of the role of concreteness in word memory^81–86^. When we considered all target word properties and searched for the optimal model to explain word memory in Experiments 2 and 3, emotional consistency and concreteness (but not semantic centrality) remained in all optimal models. This indicates that emotional consistency is not redundant with other lexical-semantic factors and continues to account for variance in memory performance when these factors are jointly modeled. Moreover, the fact that semantic centrality did not survive in three out of five models indicates that its contribution to word memorability can be substituted by concreteness, emotional consistency, and/or frequency. However, it should be noted that the semantic centrality modeled in our study was derived from word embeddings, which has the advantage of being able to provide similarity metrics for arbitrary word pairs but may not be the most accurate in capturing subjective word similarity. It is also worth noting that although semantic centrality is moderately correlated with frequency and concreteness in our dataset (see Supplementary Result S2), these variables do not exhibit multicollinearity. Future studies could use a small set of word pairs and directly measure their semantic similarity via human participant ratings, and test whether semantic centrality estimated from human similarity ratings and concreteness explain similar or unique variances. Lastly, although phonological and orthographic similarity could affect word memory, our analyses did not explicitly include such metrics beyond ensuring unique Pinyin per word. We acknowledge this as a reasonable future direction; however, it does not alter the interpretation of the current findings, which focus on semantic and lexical contributions to word memorability.

Our intrusion error analyses demonstrated that, in all three experiments, higher semantic centrality was associated with a higher likelihood of committing an intrusion error. A similar finding was also reported in Xie et al.^9^ although, notably, in the context of observing a positive relationship between semantic centrality and word memory (i.e., the opposite to that observed here). This perhaps suggests that differing mechanisms contribute to successful recall compared to associative memory errors and in accordance with this possibility, the emotion-related findings in Experiments 2 and 3 with respect to intrusion errors did not converge fully with those pertaining to successful recall. Specifically, while broad and positive emotional consistency conferred a greater likelihood of successful recall, narrow and positive emotional consistency (but not broad and negative) conferred a higher frequency of intrusion errors. Given the relatively limited sample size in our study, however (only data from participants who had committed at least 10 intrusion errors were included for this analysis), these results should be treated with caution.

Finally, it is important to consider whether word memory as assessed with a cued recall paradigm here reflects word memorability more generally, such as word memorability in free recall. While free recall and cued recall share core mnemonic mechanisms, they differ in retrieval demands, with cued recall engaging guided associative retrieval^87–91^. Indeed, the split-half consistency of target memorability in our experiments was lower than that typically observed in free recall^92^, and cue memorability showed reliable consistency, underscoring the critical role of cue information in cued recall. On the other hand, theories of episodic memory such as SAM (Search of Associative Memory)^17,93–95^ argues that all memories are associative in nature when contextual cues are taken in account. Therefore, cued recall (and associative memory paradigms more broadly) can, in our opinion, provide a theoretically meaningful perspective on memorability. Our exploratory finding that both target and cue semantic centrality predicted memory performance further highlights the associative nature of memory processing in cued-recall. Put together, free and cued recall offer complementary views of word memorability, and future research should examine whether the semantic attributes contributing to word memorability are task-general or task-specific.

In conclusion, Chinese words with weaker semantic connections to other words were found to be more memorable, while positive-positive and non-neutral word pairs demonstrated a memory advantage over their counterparts. These two attributes - semantic and emotional - seem to explain word memory in an independent and non-interactive way. These findings advance our understanding of the factors that contribute to word memorability and encourage further studies on word associative memory.

## Supplementary Information

Below is the link to the electronic supplementary material.

Supplementary Material 1

## Data Availability

Data, analysis codes, and stimuli for all experiments are available at osf.io/7bhgk/.
