# Supplementary material for: Semantic centrality and emotional valence contribute to word memorability in an associative memory task for Chinese words
Source: Sci Rep. 2026 Feb 25;16:11047. doi: 10.1038/s41598-026-37531-w (PMC13043692; doi:10.1038/s41598-026-37531-w)
Supplement: Supplementary file 1 — Supplementary Material 1 [file 41598_2026_37531_MOESM1_ESM.docx]

**Semantics and Emotion Contribute to Word Memorability in an Associative Memory Task for Chinese Words**

Zhang Allen Haoyu^1,2,*^, Wilma A. Bainbridge^3,4^, Pei Sun^2,5^, and Andy C. H. Lee^1,6^

^1^ Department of Psychology (Scarborough), University of Toronto, Toronto, Canada

^2^ Department of Psychological and Cognitive Sciences, Tsinghua University, Beijing, China

^3^ Department of Psychology, University of Chicago, Chicago, US

^4^ Neuroscience Institute, University of Chicago, Chicago, US

^5^ Faculty of Health and Wellness, City University of Macau, Macau SAR, China

^6^ Rotman Research Institute, Baycrest Centre, Toronto, Canada

^*^ For correspondence: [haoyuallen.zhang@utoronto.ca](mailto:haoyuallen.zhang@utoronto.ca)

**Table of contents**

**Result S1**. Semantic Centrality Consistency Across Models 3

**Result S2**. Correlations between Semantic Centrality, Concreteness, and Frequency 4

**Table S1.** Post-Hoc Power Estimation for All Experiments 5

**Table S2.** Single EV GLMM Results for Cue Word Properties in Experiment 1 6

**Table S3.** Single EV GLMM Results for Cue Word Properties in Experiments 2&3 7

**Table S4.** Parsimonious Model Selection for Fixed Effects: Target Semantic Centrality, Cue

Semantic Centrality, Emotional Consistency, and Interactions 8

**Table S5.** GLMM Fixed Effect Results for Cue Valence Category x Target Valence Category on

Predicting Memory Performance 9

**Result S1.** Semantic Centrality Consistency Across Models

To evaluate the consistency of semantic centrality across embedding choices, we calculated semantic centrality values for each word using two models. First, we applied the word Chinese word2vec embeddings detailed in our Methods. Second, we obtained the English GloVe embeddings as in (Xie et al., 2020). Because the GloVe lexicon is English, we translated each Chinese word into its English equivalent before extracting its embedding. Most Chinese words corresponded to a single English word with a unique GloVe embedding. However, twelve words in each of our 300-neutral-noun and 300-emotional-noun sets yielded two-word translations and were therefore excluded from this analysis.

We then calculated Spearman’s rank-order correlations between the two semantic centrality estimates for each stimulus set. Results revealed a significant positive correlation for both sets (Neutral-nouns: *r_s_* (287) = .34, *p* < .001; Emotional-nouns: *r_s_* (287) = .22, *p* < .001). These findings indicate a modest but reliable cross-model consistency in semantic centrality.

**Result S2.** Correlations between Semantic Centrality, Concreteness, and Frequency

For the set of neutral, highly concrete, nouns in Experiment 1, semantic centrality of a word was positively correlated with concreteness (*r*(299) = .33, *p* < .001). In contrast, semantic centrality was not significantly correlated with word frequency (*r*(299) = .04, *p* = .465). As a reminder, for Experiment 1, the word set was strongly restricted in its concreteness range, in keeping with (Xie et al., 2020), and semantic centrality remained a significant predictor of memory performance when control variables were considered.

For Experiments 2 and 3, semantic centrality was not retained in the final parsimonious mixed-effect model in three out of four analyses of memory performance. For this word set consisting of positive, negative, and neutral nouns, semantic centrality showed moderate and statistically significant correlations with both concreteness (*r*(299) = -.36, *p* < .001) and frequency (*r*(299) = .26., *p* < .001). A similar correlation pattern was observed when positive, negative, and neutral nouns were analyzed separately (Concreteness: *rs*(99) = -.35, -.45, -.37, *ps* < .001; Frequency: *rs*(99) = .28, .20, .35, *ps* = .004, .045, <.001, for positive, negative, and neutral nouns respectively).

**Table S1.** Post-Hoc Power Estimation for All Experiments.

| Experiment | EV | Post-Hoc Power^1^ | |
| --- | --- | --- | --- |
| Experiment 1 | Semantic centrality | 0.943 | |
| Experiment 2 | Semantic centrality | 0.987 | |
|  | Broad emotional consistency | 0.840 | |
|  | Positive emotional consistency | 0.757 | |
| Experiment 3 | Semantic centrality | 0.722 | |
|  | Broad emotional consistency | 0.807 | |
|  | Positive emotional consistency | 0.707 | |
| ^1^ Post-hoc power was estimated using the R function *mixedpower* (steps = 60, critical_value = 2, n_sim = 5000). | | |  |

**Table S2.** Single EV GLMM Results for Cue Word Properties in Experiment 1.

| EV | Random effects | χ^2^ | df | *p* | $\hat{\beta}$ | SE |
| --- | --- | --- | --- | --- | --- | --- |
| Semantic Centrality | (1+EV\|subID) + (1\|cueID) | 13.499^***^ | 1 | <.001 | -0.158 | 0.041 |
| Concreteness | (1+EV\|subID) + (1\|cueID) | 0.231 | 1 | .631 | -0.020 | 0.041 |
| Frequency | (1+EV\|subID) + (1\|cueID) | 3.845^*^ | 1 | .050 | -0.084 | 0.041 |
| Valence | (1\|subID) + (1\|cueID) | 0.189 | 1 | .664 | 0.016 | 0.037 |
| Arousal | (1\|subID) + (1\|cueID) | 0.809 | 1 | .368 | 0.034 | 0.037 |
| Age of Acquisition | (1\|subID) + (1\|cueID) | 2.830 | 1 | .093 | 0.064 | 0.037 |
| Number of Strokes | (1\|subID) + (1\|cueID) | 4.081^*^ | 1 | .043 | 0.077 | 0.038 |
| ^*^: *p* < .05; ^**^: *p* < .01; ^***^: *p* < .001. subID: subjectID. Interestingly, the cue word’s frequency and number of strokes influenced memory performance in a direction opposite to that of the target word. Specifically, cues with lower frequency and a larger number of strokes were associated with better memory performance. This pattern - contrary to the trend (significant or not) observed for target words - may reflect the fact that cues are presented during the recall phase, whereas targets are absent from perception and require active memory search. | | | | | | |

**Table S3.** Single EV GLMM Results for Cue Word Properties. For χ^2^, *p*, $\hat{\beta}$, SE, left value = Experiment 2, right value = Experiment 3.

| EV | χ^2^ | df | *p* | $\hat{\beta}$ | SE |
| --- | --- | --- | --- | --- | --- |
| Semantic Centrality | 29.259^***^; 25.401^***^ | 1 | <.001; <.001 | -0.227; -0.194 | 0.039; 0.038 |
| Concreteness | 33.302^***^; 40.220^***^ | 1 | <.001; <.001 | 0.250; 0.271 | 0.039; 0.038 |
| Frequency | 6.789^**^; 2.885 | 1 | .009; .089 | -0.110; -0.069 | 0.041; 0.041 |
| Valence | 2.101; 0.306 | 1 | .147; .580 | 0.057; 0.022 | 0.039; 0.039 |
| Arousal | 2.416; 4.716^*^ | 1 | .120; .030 | -0.062; -0.085 | 0.040; 0.039 |
| Age of Acquisition | 1.800; 5.288^*^ | 1 | .180; .021 | -0.053; -0.095 | 0.039; 0.041 |
| Number of Strokes | 0.013; 0.061 | 1 | .908; .806 | 0.005; 0.010 | 0.039; 0.041 |
| ^*^: *p* < .05; ^**^: *p* < .01; ^***^: *p* < .001  We observed that cue-level predictors (cue semantic centrality and cue concreteness) had larger coefficients than their target-level counterparts. This difference is difficult to interpret directly because cues are perceptually available during retrieval whereas targets are absent, making cue effects partly attributable to perceptual processing rather than memory strength *per se*. | | | | | |

**Table S4.** Parsimonious Model Selection for Fixed Effects: Target Semantic Centrality, Cue Semantic Centrality, Emotional Consistency, and Interactions

| Experiment | Emotional Consistency | Fixed Effects^1^ |
| --- | --- | --- |
| Experiment 1 | N/A | Target semantic centrality and cue semantic centrality |
| Experiment 2 | Broad | Emotional consistency, target semantic centrality, and cue semantic centrality |
|  | Positive | Emotional consistency, target semantic centrality, and cue semantic centrality |
| Experiment 3 | Broad | Emotional consistency, target semantic centrality, cue semantic centrality, and interaction term between target and cue semantic centrality ^2^ |
|  | Positive | Emotional consistency, target semantic centrality, cue semantic centrality, and interaction term between target and cue semantic centrality ^3^ |

^1^ The purpose of this analysis was to examine whether the cue and target semantic centrality are non-redundant predictors for memory performance. The analysis method was identical to the dual EV model (see Table 5), with the exception of additionally including cue semantic centrality and corresponding interaction terms.

^2^ Interaction term: $\hat{\beta}$ = 0.065, *p* = .015. The coefficients of semantic centrality main effects were considerably larger (target semantic centrality: $\hat{\beta}$ = -8.153, *p* = .008; cue semantic centrality: $\hat{\beta}$ = -17.873, *p* < .001).

^3^ Interaction term: $\hat{\beta}$ = 0.065, *p* = .014. The coefficients of semantic centrality main effects were considerably larger (target semantic centrality: $\hat{\beta}$ = -6.953, *p* = .026; cue semantic centrality: $\hat{\beta}$ = -16.606, *p* < .001).

**Table S5.** GLMM Fixed Effect Results for Cue Valence Category x Target Valence Category on Predicting Memory Performance. For $\hat{\beta}$, SE, *p*, left value = Experiment 2, right value = Experiment 3.

| EV^1^ | $\hat{\beta}$ | SE | *p* |
| --- | --- | --- | --- |
| cue_salient | 0.010; 0.036^*^ | 0.020; 0.018 | .596; .049 |
| cue_positive | 0.085^*^; 0.036 | 0.034; 0.032 | .012; .267 |
| target_salient | 0.052^*^; 0.041 | 0.024; 0.025 | .030; .098 |
| target_positive | 0.088^*^; 0.033 | 0.042; 0.043 | .037; .449 |
| cue_salient : target_salient | 0.028^*^; 0.011 | 0.014; 0.013 | .045; .382 |
| cue_positive : target_salient | -0.014; -0.041 | 0.024; 0.023 | .541; .074 |
| cue_salient : target_positive | 0.009; 0.051^*^ | 0.024; 0.022 | .722; .024 |
| cue_positive : target_positive | 0.004; 0.049 | 0.042; 0.039 | .922; .211 |
| ^*^: *p* < .05  ^1^ We defined two theoretically meaningful contrasts for the valence category variables. Specifically, the *salient* contrast (Negative = 1, Neutral = -2, Positive = 1) compares emotionally salient words (positive and negative) against neutral words, whereas the *positive* contrast (Negative = -1, Neutral = 0, Positive = 1) compares positive versus negative words. | | | |
